# Authors’ Response to Peer Reviews of “Effects of Interventions for the Prevention and Management of Maternal Anemia in the Advent of the COVID-19 Pandemic: Systematic Review and Meta-Analysis”

**DOI:** 10.2196/81711

**Published:** 2025-10-06

**Authors:** John Kyalo Muthuka, Dianna Kageni Mbari-Fondo, Francis Muchiri Wambura, Kelly Oluoch, Japheth Mativo Nzioki, Everlyn Musangi Nyamai, Rosemary Nabaweesi

**Affiliations:** 1 Department of Community Health and Health Promotion, Faculty of Public Health, Kenya Medical Training College, Mbagathi Way, Kenyatta National Hospital, Nairobi, 30195-00100, Kenya, 254 724274843; 2 Epidemiology/Public Health Section, KEMRI Graduate School of Health Sciences, Kenya Medical Research Institute, Nairobi, Kenya; 3 Alberta Health Services, Edmonton, AB, Canada; 4 Kenya Medical Training College, Nairobi, Kenya; 5 School of Nursing, Andrews University, Berrien Springs, MI, United States; 6 School of Global Health, Meharry Medical College, Nashville, TN, United States

**Keywords:** maternal anemia, anemia in pregnancy, COVID-19, pregnancy complications, meta-analysis, maternal and child health, anemia prevention, reproductive health

*This is the authors’ response to peer-review reports for “Effects of Interventions for the Prevention and Management of Maternal Anemia in the Advent of the COVID-19 Pandemic: Systematic Review and Meta-Analysis.”*

## Round 1 Review

### Anonymous [1]

*1. Retrospective studies: The majority of the studies included in the meta-analysis [2] were retrospective epidemiological studies, which may have limitations in terms of bias, data accuracy, and causality compared to prospective studies or randomized controlled trials. This could affect the reliability and generalizability of the findings.*

**Response:** These were the only available studies at the time. However, subgroup analysis was conducted to address these limitations and enhance the validity of the findings and conclusions.

*2. Heterogeneity: The high heterogeneity identified in the pooled effect estimates suggests variability in study designs, interventions, and outcomes across the included studies. This heterogeneity can impact the interpretation of the results and the ability to draw consistent conclusions.*

**Response:** As highlighted by other reviewers, this issue has been addressed comprehensively.

*3. Publication bias: The presence of publication bias indicated by the asymmetrical funnel plot could introduce bias in the pooled effect estimates. This bias may be due to the selective reporting of studies with significant results, potentially skewing the overall findings.*

**Response:** This has been acknowledged as a limitation and reported in the study along with measures that were implemented to mitigate it.

*4. Limited scope: Some studies may not have clearly defined the age range of participants or the specific stage of the gestation period analyzed. A lack of detailed information on these aspects could limit the applicability and generalizability of the results to specific subgroups of pregnant women.*

**Response:** While this limitation was noted, the study primarily focused on the types of interventions used during COVID-19 and how they impacted the norm in managing maternal anemia. The scope was broadly centered on reproductive age but not stratified into specific age groups.

*5. Indirect effects of COVID-19: While the study focused on the direct impact of COVID-19 on maternal anemia interventions, indirect contributions of the pandemic on anemic conditions may not have been fully elucidated. Understanding these indirect effects could provide a more comprehensive view of the challenges faced during the pandemic.*

**Response:** Subgroup and sensitivity analysis were conducted to explore the perceived indirect effects. The variability in studies likely included indirect effects, which have been explained as a potential limitation.

*6. Effectiveness trends: The decreasing trend in the effectiveness of interventions against maternal anemia from 2020 to 2022 raises questions about the sustainability and adaptability of intervention strategies, especially in the context of global health emergencies. Further research is needed to explore the reasons behind this trend and potential strategies for improvement.*

**Response:** Further research is recommended to investigate the reasons behind this trend and to develop strategies for improvement, as suggested in the Feasible Policy Recommendations section.

### Reviewer IG [3]

#### Major Comments

*1. Scientific rigor and novelty.*

*Strength: The focus on maternal anemia interventions during COVID-19 is unique and addresses a significant gap in the literature.*

*Issue: The study does not establish the novelty of its findings clearly. It cites several similar meta-analyses but does not differentiate its contribution.*

*Recommendation: Clarify how this meta-analysis advances existing knowledge. Are there new methodologies, expanded datasets, or novel insights?*

**Response:** This meta-analysis advances existing knowledge through rigorous methodologies and expanded datasets from 11 articles with 6129 participants. It reveals new insights, including a 39% utility in preventing and managing maternal anemia, with significant impacts from education (28%), medicinal administration (19%), iron supplementation (17%), and intravenous ferric carboxylmaltose (15%). It highlights regional differences, particularly higher effectiveness in Africa, and underscores the need for multicenter studies and ongoing research.

*2. Study design and methodology.*

*Inclusion criteria: The inclusion of preprints and unpublished data raises concerns about the reliability and quality of the evidence.*

*Suggestion: Clearly discuss the rationale for including preprints and outline strategies to mitigate biases.*

*Subgroup analysis: While subgroup analyses are insightful, the interpretation of heterogeneity (I²>90% in multiple cases) is not adequately addressed. The sensitivity analyses seem to mitigate this but are not discussed in sufficient depth.*

*Suggestion: Incorporate a robust discussion of the potential sources of heterogeneity and its implications for the results.*

**Response:** Inclusion criteria: While it was initially planned, preprints ultimately were not included, resolving this issue.

Subgroup analysis: Several factors contributed to this heterogeneity. Retrospective epidemiological studies dominated, with only 4 randomized controlled trials providing more robust evidence. Variability in participant age ranges and gestation stages, coupled with COVID-19’s indirect effects on hemoglobin levels, contributed to this limitation. These issues are explained in detail, emphasizing the need for future research on pandemics and disasters.

*3. Data presentation.*

*Tables and figures: Tables and figures are overly complex and lack clarity.*

*Suggestion: Simplify forest and funnel plots for better readability. Ensure that all figures are annotated clearly.*

*Forest plots: Some rate ratio confidence intervals (eg, in subgroup analysis) overlap with no-effect lines, which undermines conclusions about statistical significance.*

*Suggestion: Address these overlaps explicitly in the Discussion.*

**Response:** Tables and figures: Figures have been appropriately annotated and simplified to improve readability, as generated by the software.

Forest plots: This issue has been addressed in the Discussion, aligning with the statistical outputs.

*4. Statistical analysis.*

*Publication bias: The funnel plots indicate substantial publication bias. This is acknowledged but inadequately addressed in the Discussion.*

*Suggestion: Include a deeper discussion of how this bias impacts the reliability of pooled estimates.*

*Fixed- versus random-effects models: The rationale for choosing fixed- or random-effects models for different analyses is not well-articulated.*

*Suggestion: Explain this choice clearly, especially in the context of high heterogeneity.*

**Response:** Publication bias: Publication bias likely exaggerated intervention effectiveness, potentially skewing results and conclusions. Nevertheless, comprehensive searches, statistical adjustments, and inclusion of the most relevant studies at the time ensured accurate and reliable meta-analysis outcomes, enhancing study validity. Fixed- versus random-effects models: Both models were used to balance within-study and between-study variability. This dual approach strengthened the robustness and credibility of the findings, ensuring accurate pooled estimates for maternal anemia interventions.

*5. Interpretation of results.*

*The interpretation of intervention effects (eg, a 17% improvement for iron supplementation) does not account for clinical significance, which may differ from statistical significance.*

*Suggestion: Discuss the practical implications of these findings, especially in low-resource settings.*

**Response:** The findings highlight that interventions like iron supplementation, education, and medicinal administration are critical for improving maternal anemia outcomes, particularly in low-resource settings. Even modest improvements significantly benefit maternal and infant health by reducing complications during pregnancy and childbirth.

6. Language and readability.

*The manuscript is riddled with grammatical errors, unclear phrasing, and redundancies. For instance:*

*“The effect on prevention, control, management and or treatment of anemia was calculated and compared between the intervention and the comparator arms.”*

*Suggestion: Simplify and clarify language to improve readability.*

*Acronyms (eg, RR, CI, IFA) are used without clear explanation.*

*Suggestion: Ensure all acronyms are defined upon first use.*

**Response:** Grammar, phrasing, and redundancies have been thoroughly refined to enhance readability throughout the document. All acronyms have been defined upon their first use.

*7. Ethical considerations.*

*The manuscript mentions that some data are unpublished. It is unclear whether these studies adhered to ethical guidelines.*

*Suggestion: Add a section on ethical considerations, particularly around the inclusion of unpublished studies.*

**Response:** Only studies published in English between December 2019 and August 2022 were included, ensuring ethical considerations were met.

*8. Discussion and Conclusion.*

*Weakness: The Discussion is repetitive and does not critically engage with the limitations of the study or the broader implications of the findings.*

*Suggestion: Provide a more focused discussion of limitations (eg, high heterogeneity, reliance on observational studies), implications for practice and policy, and recommendations for future research.*

#### Minor Comments

*1. Abstract.*

*Issue: The abstract lacks precision and overuses vague terms (eg, “several anemia interventions”).*

*Suggestion: Summarize key findings clearly, avoiding overgeneralizations.*

**Response:** Key findings have been summarized clearly, avoiding generalizations. The introduction has been streamlined to focus on the problem, knowledge gaps, and study objectives.

*2. Introduction.*

*The Introduction is overly lengthy and includes redundant information (eg, definitions of anemia repeated multiple times).*

*Suggestion: Streamline the Introduction to focus on the problem, the gap in knowledge, and the study’s objectives.*

*3. References.*

*References are incomplete and inconsistently formatted.*

*Suggestion: Ensure all references follow a standardized format (eg, APA, AMA).*

**Response:** References have been corrected and formatted in the Vancouver style.

*4. Figures.*

*Figures are not numbered or titled appropriately.*

*Suggestion: Include clear figure numbers, titles, and legends for all figures.*

**Response:** All figures have been numbered, titled, and provided with legends for clarity.

#### Recommendations for Authors

*Based on the above assessment, this manuscript requires major revisions. Key issues include addressing heterogeneity and publication bias in statistical analysis, improving clarity and rigor in data presentation, and enhancing language and readability.*

**Response:** These revisions have been implemented throughout the document.

### Reviewer JS [4]

#### Major Comments

*1. There is no conclusion in this manuscript. Add a Conclusion section that summarizes the content of this study.*

**Response:** A Conclusion section has been added at the end of the write-up, summarizing the study’s key content and findings.

#### Minor Comments

*2. Introduction section. Briefly explain the types of interventions implemented during COVID-19 to prevent anemia in pregnant women. Provide a brief explanation of the differences in anemia prevention interventions before and after COVID-19.*

**Response:** The introduction has been updated to include this information: "During the COVID-19 pandemic, interventions adapted to include telemedicine, remote consultations, and increased community health worker involvement to address health care disruptions [5-8]. These measures aimed to ensure continued support for pregnant women [9,10]. Interventions to prevent anemia in pregnant women included iron and folic acid supplementation, dietary modifications, education and awareness programs, telemedicine, and remote consultations, as well as community-based interventions [11,12].”

*3. Discussion, at the end of the Discussion section. Include the main findings of this study and emphasize their significance in addressing anemia-related challenges. Highlight the contribution of the study results to public health, particularly how the findings can inform or improve anemia prevention and treatment strategies in health care systems. Provide practical recommendations or actionable steps based on the study’s outcomes that can be implemented in maternal health care policies and programs.*

**Response:** The main findings have been incorporated into the Discussion. The meta-analysis advances knowledge by using rigorous methodologies and expanded datasets from 11 articles involving 6129 participants. It reveals new insights, including a 39% utility in preventing and managing maternal anemia. The analysis highlights the effectiveness of education (28%), medicinal administration (19%), iron supplementation (17%), and intravenous ferric carboxylmaltose (15%). Additionally, regional differences, with higher effectiveness noted in Africa, underscore the importance of multicenter studies and ongoing research.

Public health: The findings demonstrate that unforeseen pandemics may compromise anemia control, affecting interventions for maternal anemia. It is essential to screen pregnant women to identify the best intervention options for achieving optimal outcomes during crises. The impact of the COVID-19 pandemic on anemia interventions should be further studied. Key health care stakeholders must address risks posed to maternal anemia management outcomes. Future research should explore mechanisms that drive or reduce the risks of compromised maternal anemia interventions.
